# Paradigms of frustration in superionic solid electrolytes

**DOI:** 10.1098/rsta.2019.0467

**Published:** 2021-11-29

**Authors:** Brandon C. Wood, Joel B. Varley, Kyoung E. Kweon, Patrick Shea, Alex T. Hall, Andrew Grieder, Michael Ward, Vincent P. Aguirre, Dylan Rigling, Eduardo Lopez Ventura, Chimara Stancill, Nicole Adelstein

**Affiliations:** ^1^ Laboratory for Energy Applications for the Future and Materials Science Division, Lawrence Livermore National Laboratory, 7000 East Avenue, Livermore, CA 94550, USA; ^2^ Department of Chemistry and Biochemistry, San Francisco State University, San Francisco, CA, USA

**Keywords:** superionic, *ab initio* molecular dynamics, solid electrolyte, frustration

## Abstract

Superionic solid electrolytes have widespread use in energy devices, but the fundamental motivations for fast ion conduction are often elusive. In this Perspective, we draw upon atomistic simulations of a wide range of superionic conductors to illustrate some ways frustration can lower diffusion cation barriers in solids. Based on our studies of halides, oxides, sulfides and hydroborates and a survey of published reports, we classify three types of frustration that create competition between different local atomic preferences, thereby flattening the diffusive energy landscape. These include chemical frustration, which derives from competing factors in the anion–cation interaction; structural frustration, which arises from lattice arrangements that induce site distortion or prevent cation ordering; and dynamical frustration, which is associated with temporary fluctuations in the energy landscape due to anion reorientation or cation reconfiguration. For each class of frustration, we provide detailed simulation analyses of various materials to show how ion mobility is facilitated, resulting in stabilizing factors that are both entropic and enthalpic in origin. We propose the use of these categories as a general construct for classifying frustration in superionic conductors and discuss implications for future development of suitable descriptors and improvement strategies.

This article is part of the Theo Murphy meeting issue ‘Understanding fast-ion conduction in solid electrolytes’.

## 1. Introduction

Superionic conductors are materials—typically ionic solids—that feature rapid ion conductivity approaching or even exceeding that of liquids. Potential applications are diverse, encompassing fuel cells, coatings, membranes, solid-state hydrogen storage and electrical energy storage [1–4]; however, ${\mathrm{Li}}^{+}$ and ${\mathrm{Na}}^{+}$ conductors in particular have garnered significant recent attention for their potential use in solid-state batteries [5–7]. Although the phenomenon is relatively rare, fast cation conductors can be found across several classes of materials, including oxides, sulfides, halides and structures featuring various types of polyatomic anions. Superionic behaviour has also been observed in materials with a variety of different crystallographic symmetries, as well as in glassy and interface-dominated composite materials [7]. Much is known regarding the mechanisms of ion conduction in specific materials, and several universal descriptors for superionic activity have recently been proposed or employed [1,3,5,6,8–11]. Nevertheless, exceptions to these rules are numerous, suggesting much remains to be understood. In addition, these studies employ varying terminology, motivating the need for a common language for describing the underlying physics.

**Figure 1.**
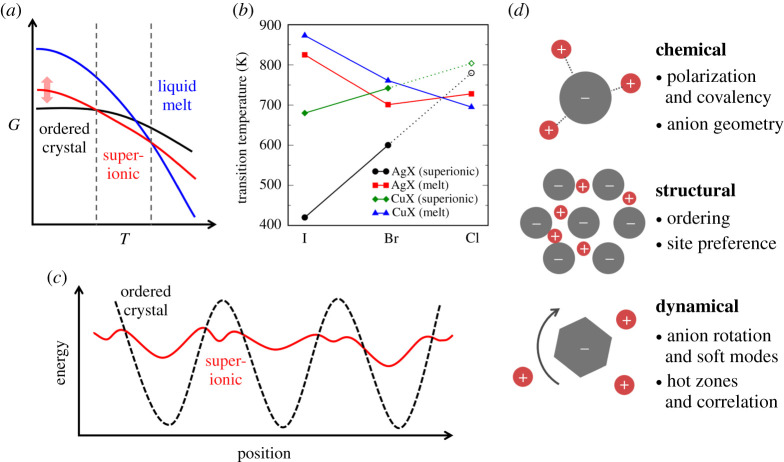
Frustration and flattened energy landscapes. (*a*) Whereas the thermodynamics of ordered crystals are conventionally dominated by enthalpy and liquids by entropy, superionic phases have significant contributions from both factors and are stabilized at temperatures below the melting point if the configurational entropy is sufficient. (*b*) Superionic and melting transition temperatures for silver and copper halides, illustrating the entropic competition between the two as the chemistry is changed. Dashed lines and open symbols are linear extrapolations. (*c*) Schematic of a diffusive energy landscape for a conventional crystal (dashed black line) versus a crystal with a frustrated energy landscape that is flattened (solid red line). (*d*) Illustration of the three types of frustration covered in this Perspective: chemical, structural and dynamical. (Online version in colour.)

Many of the proposed descriptors are connected to the formation of diffusive energy landscapes that are intrinsically flattened compared to conventional solids. Such landscapes are counterintuitive to our conventional understanding of crystalline ionic solids, the stability of which is generally connected to periodic arrays of alternating cations and anions that rely on enthalpic stabilization by strong Coulomb interactions. Instead, superionic materials phases are stabilized not only enthalpically, but also entropically. In this way, they sit at the thermodynamic boundary between conventional solids and entropy-stabilized liquids, relying on a liquid-like diffusive sublattice within a solid matrix (figure 1*a*). Consequently, many superionic materials feature relatively small latent heats of melting. Whether or not these entropically stabilized solid superionic phases manifest depends on their competition with liquid melting, which naturally results in a larger entropy increase at the expense of the enthalpy of cohesion. This picture suggests that many ionic solids may have possible superionic phases, but that these phases are unstable or metastable with respect to the melt.

Figure 1*b* illustrates the competition between the liquid melt and superionic phase for the case of the silver and copper halides, which have been studied extensively as archetypes of fast ion conduction [2,12–15]. The superionic phases of these materials have intrinsic vacancies and highly mobile cations. The iodides exhibit the fastest diffusion and hence highest configurational entropy, which is the dominant contributor to entropic stabilization [2,16,17]. The first-order superionic phase transitions occur for AgI, CuI and CuBr, whereas AgBr exhibits superionic behaviour in the rocksalt structure at temperatures approximately above 600 K. On the other hand, AgCl and CuCl melt before this highly conductive phase is stabilized. These differences can be rationalized in terms of figure 1*a*,*b*: as the halide size and polarizability increases, the conductivity improves and the configurational entropy is enhanced. This entropy lowers the free energy curve associated with the superionic phase, decreasing the superionic transition temperature while increasing the melting temperature. For bromides, the configurational entropy is much reduced, and the free energy curve for the superionic phase is raised. For instance, the superionic α-CuBr phase is only stable over a narrow 20 K range as the superionic transition increases and the melting temperature decreases. Finally, for the chlorides, the configurational entropy of the superionic phases is insufficient to overcome the melt (the dashed lines in figure 1*b* show hypothetical extrapolations for the expected superionic transition temperatures). These trends highlight not only the importance of entropy, but also its critical connection to the cation–anion chemistry and structure.

In superionic solids, the high configurational entropy derives in large part from a flattened diffusive energy landscape featuring a high density of local minima (figure 1*c*). To better understand superionic behaviour and devise improved descriptors for the discovery and optimization of new solid electrolytes, it is of paramount importance to understand the physical factors that generate these local minima. To accommodate the local concentration fluctuations that drive long-range ion diffusion, the materials must tolerate a unusual degree of local deviation from ideal charge balance. As a result, other factors beyond global electrostatic point–charge interactions (i.e. Madelung energy) can play a significant role. These other physicochemical factors, which typically reflect local energetic preferences, can help to accommodate spatio-temporal deviations in ion arrangements. Such competition between conventional point-charge electrostatics and other local factors governing the cation–anion interaction is an example of *frustration* [18,19], whereby static ion patterning into the deep electrostatic energy minima seen in typical ionic crystals is prevented by an inability to simultaneously accommodate all local and global energetic preferences.

In practice, this frustration can arise from a variety of sources. In this Perspective, we revisit three categories of frustration classified in our previous work on *closo*-borate materials [20–22] (figure 1*d*), clarifying our earlier definitions and broadening the scope to include examples from several other superionic conductors studied by our team and by others in the solid-state electrolyte community. In each case, the connection to fast ion mobility is discussed in detail. Our intention is to elaborate upon some of the universal motivations for unusually low activation barriers for diffusion in superionic conductors, many of which are also echoed in a series of excellent reviews and descriptor-focused investigations over the past two decades [1,3,5–11,14,19,23–29]. We further suggest that the categories of frustration presented here may be used as a universal framework for classifying superionic solids and emerging design strategies.

We refer to the first category as *chemical frustration*, which derives from a competition between the global Madelung energy derived from classical Coulomb interactions and local factors such as covalency, polarization and local ion clustering. We call the second category *structural frustration*, which involves the inability of diffusive ions to find preferred lattice sites or to pattern onto those sites. We deem the third category *dynamical frustration*, which concerns fluctuations in the energy landscape due to soft lattice perturbations and rotations at frequencies relevant to diffusion; this is a special case of the first two types of frustration, except in the temporal domain. Focusing on crystalline cation conductors, we employ molecular dynamics simulations from our own studies and discussions of complementary computational studies by other groups to show examples of each of these types of frustration in multiple types of solid electrolyte materials. Ultimately, the literature references are compiled into a proposed classification, from which we conclude that there is no single driver of energy landscape flattening, but rather that different classes of fast ion conductors and design strategies are grounded in different physical frustration origins.

## 2. Chemical frustration

### (a) Polarization and covalency

For many superionic cation conductors, it has long been suggested that a key driver of fast ion migration lies in the specific interaction between the mobile cations and the lattice-forming anions [2,30–32]. Within our definition, ‘chemical frustration’ can occur if this interaction involves a competition between two or more physico-chemical ingredients in the nature of the anion–cation bond. A classic example of this frustration involves a contest between ionic and covalent preferences that motivate different preferred local structural environments that cannot easily be accommodated simultaneously. For instance, the competition between ionic and partial-covalent preferences has been cited in the context of silver and copper superionic conductors, given the known ability of these elements to form partially covalent bonds [13,31,33,34]. The presence of a degree of covalency in these materials is further suggested by the strong local coordination environment that is retained by the cations surrounding the anions. Although this variety of chemical frustration manifests most strongly with mobile d-electron cations, our own research suggests that covalent bond character is leveraged for frustration in a wider variety of cation conductors paired with large anions [35], which have charge distributions that can easily deform and polarize. Nevertheless, we emphasize that the presence of covalent character in the cation–anion interaction alone is insufficient for chemical frustration, as this is common; rather, the frustration arises from the *competitive coexistence* of both ionic and partial covalent anion–cation interactions.

*Ab initio* molecular dynamics (AIMD) simulations have been employed to give a more complete and quantitative picture of this competitive coexistence, as well as to probe the effects of chemical bonding, partial covalent character and electron density distortions that are often difficult to quantify experimentally. To date, interaction of the diffusing species with the static host lattice through dynamic polarization of an anion or the breaking and forming of polar covalent bonds has been explored by the authors in α-AgI [13], ${\mathrm{Li}}_{3}{\mathrm{InBr}}_{6-x}{\mathrm{Cl}}_{x}$ [35,36], and ${\mathrm{MHCB}}_{11}H_{5}X_{6}$ (M=Li,Na; X=Cl,Br) [22]. Furthermore, research by Zeier and colleagues has hinted at the role of chemical frustration as a driver of high conductivity in argyrodites [37] and thiophosphates [38]. In argyryodites, it was reported that disorder from site exchanges between $S^{2-}$ and the ${\mathrm{Cl}}^{-}/{\mathrm{Br}}^{-}$ ions was found to promote diffusion. The authors posit that the cause is ‘possibly due to the more directional covalent character of the $S^{2-}$ anion, which leads to a large degree of fluctuating chemical environments’, very similar to the concepts discussed here [37]. Chemical frustration is also reflected in the ‘inductive effect’ invoked by Zeier *et al.* to explain how altered cation–anion interactions upon alloying of Ge and Sn can redistribute local charge and modulate barriers in ${\mathrm{Li}}_{10}{\mathrm{Ge}}_{1-x}{\mathrm{Sn}}_{x}P_{2}S_{12}$ thiophosphates [39,40]. Zhang *et al.* similarly reported that anion substitution of $O^{2-}$ for $S^{2-}$ in the thiophosphate materials ${\mathrm{Li}}_{3}{\mathrm{PS}}_{4}$ and ${\mathrm{Li}}_{10}{\mathrm{GeP}}_{2}S_{12}$ changes bond character to lower the migration barrier [41]. Although fluctuating bond character was not explicitly identified in all of these studies, the strong dependence of diffusion on the specific cation–anion bond character suggests the prominent role of chemical frustration, which could be confirmed by further polarization or covalency analysis.

**Figure 2.**
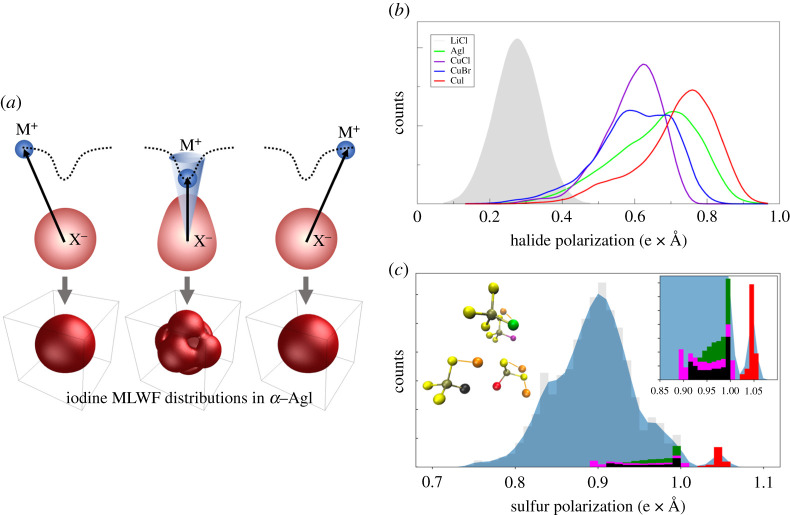
Chemical frustration via polarization and covalency. (*a*) Upper panels: ‘capture’ of a diffusing cation ($M^{+}$) by a polarizable anion ($X^{-}$), accompanied by the temporary introduction of directional covalent character in the anion–cation bond (centre). Lower panels: corresponding density isosurfaces of Maximally Localized Wannier Function centres for iodine in α-AgI, revealing fluctuations between isotropic distributions for shorter-distance, unpolarized iodine orbitals compared to directional distributions for longer-distance, polarized iodine orbitals with partial covalent character (centre). (*b*) Asymmetric distributions of anion polarization for several superionic halides sampled from the dynamics trajectories. The corresponding symmetric distribution for LiCl is shown in shaded grey for reference. (*c*) Distribution of anion polarization for LPS. The left inset shows some of the [PS_4_]^3−^ coordination environments occurring near jumping Li atoms (orange) in the 200 fs time window centred on the jump. Central P atoms are shown in grey. S atoms are shown in yellow, with the most polarized S atoms within these configurations shown in red, black, green and magenta (purple and green S atoms also share a bridging Li). Corresponding contributions of the most polarized S atoms to the upper tail of the polarization distribution are plotted in the same colour scheme and expanded in the right inset for easier viewing. Portions of panel (*a*) were adapted with permission from Wood & Marzari [13] (Copyright 2006 American Physical Society). (Online version in colour.)

One way to describe polarization and covalency within AIMD simulations is through Maximally Localized Wannier Functions (MLWFs), which provide a molecular orbital-like description of the electronic density that can be readily visualized and analysed [42]. In addition, the vector sum of the Wannier centres with respect to the atomic cores provides a quantitative measure of the polarization, which can be tracked throughout the trajectory to assess changes to bond character. MLWFs were first employed in superionic systems by Wood and Marzari to confirm fluctuating bond character between ${\mathrm{Ag}}^{+}$ and $I^{-}$ in α-AgI [13], which had been previously theorized by Aniya as a driving force for fast ion conduction [31]. The essential results, summarized in figure 2*a*, showed a coexistence between shorter- and longer-distance Wannier centres, corresponding to chiefly ionic and partial covalent Ag-I bond interactions, respectively. The spatial distributions of each type of interaction evidence the directional nature of the partial covalent bonds versus the non-directional ionic interactions. Further analysis showed that ${\mathrm{Ag}}^{+}$ ions within a certain angular and radial threshold are ‘captured’ into a partial covalent bond, causing them to stay resident for longer. Because this covalency is inherently directional, the captured ${\mathrm{Ag}}^{+}$ ions retain a well-defined angular distribution around each iodine. Above the superionic transition temperature, the system is not able to accommodate this directional preference for all ${\mathrm{Ag}}^{+}$ ions, leaving some ionically bonded ${\mathrm{Ag}}^{+}$ ions free to migrate—a characteristic signature of chemical frustration and its effect on ion mobility. In addition, the partial covalent bonds were found to break and form continually above the superionic transition temperature, leading to fluctuations in local bond chemistry that are harnessed for diffusion.

This same bond-fluctuation analysis was later applied to ${\mathrm{Li}}_{3}{\mathrm{InBr}}_{6-x}{\mathrm{Cl}}_{x}$ [43,44] to demonstrate a similar signature of bimodal coexistence of ionic and partial covalent bond character for the Li-Br interactions [35,36]. Although weaker than in α-AgI, this bond signature demonstrated that similar signatures of covalency can be detected even in ${\mathrm{Li}}^{+}$ conductors if polarizable anions are present [35], reflecting the fact that bonds between small cations and large anions can possess a degree of covalency. Further analysis of mobile ${\mathrm{Li}}^{+}$ hops confirmed that this coexistence of ionic and polar covalent bond character—and its accompanying frustration—were indeed relevant contributors to ionic mobility. We also showed that Cl/Br alloying could be used to maximize chemical frustration by properly matching anion size to facilitate simultaneous interaction with multiple ${\mathrm{Li}}^{+}$ neighbours [36].

In lieu of the full analysis of individual MLWFs, a simpler descriptor for the presence of chemical bond frustration can be found in the shape of the anion polarization distribution derived from their vector sum. Figure 2*b* shows an example for superionic α-AgI and α-CuI binary halides, as well as the superionic phase of CuBr (and a hypothetical CuCl phase) that operate at higher temperatures but are nominally less conductive than the iodide counterparts. In each case, the distribution of the halide polarization shows multimodal behaviour with distinct shoulders appearing at lower polarization, indicating the coexistence of at least two competing types of chemical interactions. Moreover, the polarization is in large part determined by the size of the anion, with the breadth and average of the distribution increasing as the anion size increases from ${\mathrm{Cl}}^{-}$ to $I^{-}$ across the copper series. Overall, the skew and multimodal character are most evident for the fastest-conducting iodides. To emphasize the contrast with non-superionic systems, we also ran AIMD on non-conductive LiCl, which shows a unimodal distribution with the expected symmetric thermal broadening. This implies that the shape of the polarization distribution could be a promising descriptor of superionic behaviour, particularly for halide systems.

A similar analysis can also be applied to sulfide conductors and polyatomic anions, such as the ${\left[{\mathrm{PS}}_{4}\right]}^{3-}$ units in superionic $\text{β-}{\mathrm{Li}}_{3}{\mathrm{PS}}_{4}$ (LPS) [7,45–47]. In order to separate the electronic polarization effects from the configurational variability, we focus on the crystalline phase of LPS and plot the polarization distribution of S atoms in figure 2*c*. The distribution likewise shows multimodal behaviour, including a distinct shoulder and second peak at higher polarization. Whereas the diffusive ions in AgI were associated with lower polarization, these high-polarization sulfurs play a more direct role in LPS. The specific connection between polarization and diffusion is shown by isolating discrete jump events and extracting the polarization contributions of key S atoms nearby the temporarily mobile ${\mathrm{Li}}^{+}$ (selected within a 200 fs window centred on the local jump event). It is clear that ${\mathrm{Li}}^{+}$ environments that feature stronger polarization are correlated with high mobility. Further decomposition into different Li–${\mathrm{PS}}_{4}$ coordination environments (coloured bars) reveals that the strongest polarization is observed for S atoms that are bound to the same ${\left[{\mathrm{PS}}_{4}\right]}^{3-}$ complex but are not directly adjacent to ${\mathrm{Li}}^{+}$, evidencing the complexity of polarization behaviour for polyatomic anions. Some specific features of chemical frustration in polyatomic anions are discussed further in the next section.

One consequence of fluctuations in polarization and covalent character is that multiple closely spaced local minima are introduced in the energy landscape. As discussed in the Introduction and depicted schematically in figure 1*c*, this results in highly anharmonic, broad energy wells. Interestingly, the presence of these broad wells closely echoes findings in a recent study by He *et al.* based on a survey of a large number of ionic conductors [24]. The authors found clear evidence that ${\mathrm{Li}}^{+}$ diffusivity is strongly correlated with the presence of ‘enlarged’ ${\mathrm{Li}}^{+}$ sites comprising multiple local minima in crystal structures that are known to facilitate superionic conduction. These enlarged sites are accompanied by significantly anisotropic atomic displacements that reflect a specific chemical interaction beyond point-charge electrostatics.

### (b) Polyatomic anion geometry

In the previous section, we examined the role of chemical frustration as it pertains to the distortion of the electron density around an anion due to polarization or bond covalency effects. However, for polyatomic anions, the geometry and flexibility of the atoms themselves can introduce similar effects by altering the local configurational preferences of the cations. In particular, additional chemical frustration is encountered when the symmetry of the polyatomic anion itself does not match the crystal lattice symmetry. This reflects a competition between local cation–anion interactions and global electrostatic site preferences expected within a Madelung-type description based solely on point charges.

**Figure 3.**
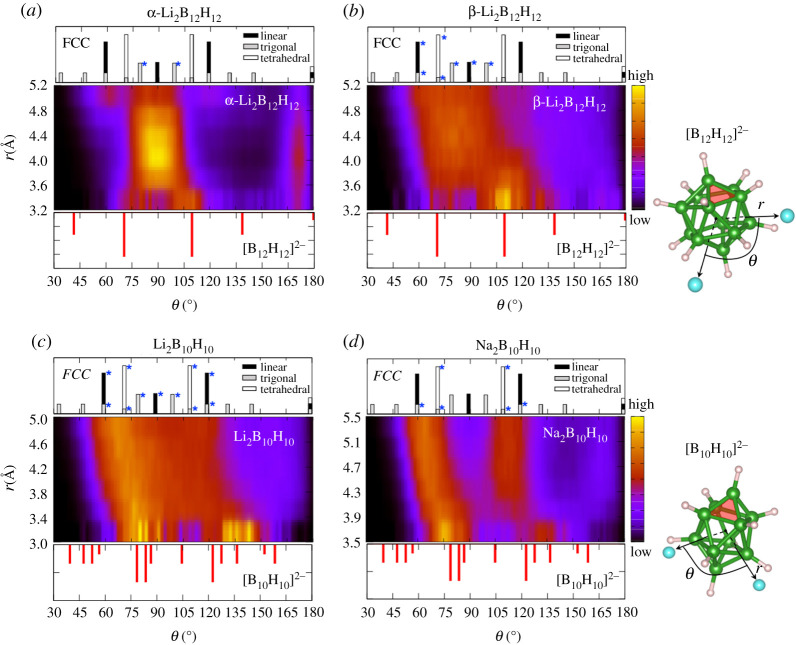
Chemical frustration via anion geometry and electrostatic preferences. Average cation–anion–cation angular distributions (θ) of ${\mathrm{Li}}^{+}$/${\mathrm{Na}}^{+}$ around *closo*-borate anions as a function of cation distance r from the anion centre in *FCC* crystals of (*a*) non-superionic $\text{α-}{\mathrm{Li}}_{2}B_{12}H_{12}$ and superionic (*b*) $\text{β-}{\mathrm{Li}}_{2}B_{12}H_{12}$, (*c*) ${\mathrm{Li}}_{2}B_{10}H_{10}$, and (*d*) ${\mathrm{Na}}_{2}B_{10}H_{10}$ at 800 K. The data are plotted within the internal reference frames of the anions. The panels above each subfigure show the angular distributions of *FCC* interstitial lattice sites, with the key sites occupied in the dynamics indicated with asterisks. The panels below each subfigure indicate the ${\left[B_{n}H_{n}\right]}^{2-}$ boron triad face centres, with the corresponding anion geometries also shown for reference. The superionic phases exhibit a continuous progression from short-ranged anion templating to longer-ranged lattice interstitial site symmetry. Portions of (*b*) were adapted with permission from Kweon *et al*. [21] (Copyright 2017 American Chemical Society). (Online version in colour.)

A prototypical example of frustration between the symmetries of the anion and the lattice can be found in the superionic *closo*-borate salts—a subclass of highly conductive hydroborate solid electrolytes based on large, cage-like anions like ${\left[B_{12}H_{12}\right]}^{2-}$ and ${\left[B_{10}H_{10}\right]}^{2-}$ [4,20,21,48–55]. Figure 3*b* shows the distribution of cation–anion–cation angles plotted as a function of distance from the anion centre within the fixed reference frame of the anion. For superionic $\text{β-}{\mathrm{Li}}_{2}B_{12}H_{12}$ or ${\left(\mathrm{Li}/\mathrm{Na}\right)}_{2}B_{10}H_{10}$ in figure 3*b*–*d*, cations at short distances are templated by the distribution of B triad docking sites on the faces of the polyatomic anion itself (${\left[B_{12}H_{12}\right]}^{2-}$ or ${\left[B_{10}H_{10}\right]}^{2-}$), where the local anion–cation interaction strength is maximal [21]. However, as cations move slightly farther from the anion, the peak of the angular distribution gradually moves to reflect the distribution of occupied interstitial sites in the *FCC* lattice (marked with asterisks in figure 3: linear, trigonal and tetrahedral sites for $\text{β-}{\mathrm{Li}}_{2}B_{12}H_{12}$ and ${\mathrm{Li}}_{2}B_{10}H_{10}$; trigonal and tetrahedral sites for ${\mathrm{Na}}_{2}B_{10}H_{10}$). The gradual progression of peak distribution from short-ranged anion templating to longer-ranged lattice interstitial site symmetry for superionic systems suggests a large overlap of potential energy surfaces for the anion-determined configurations and the lattice-determined configurations, which interchange and introduce frustration as cations vibrate within their local environments.

The competition between the anion symmetry and the lattice symmetry serves to flatten the potential energy surface and promote ion mobility [21,56]. To illustrate this causality more explicitly, our analysis in figure 3 also includes the non-superionic $\text{α-}{\mathrm{Li}}_{2}B_{12}H_{12}$ phase, which shares the same *FCC* lattice symmetry as its superionic $\text{β-}{\mathrm{Li}}_{2}B_{12}H_{12}$ cousin but features anions in fixed orientations and different interstitial site occupancy. As shown in figure 3*a*, the lattice symmetry of $\text{α-}{\mathrm{Li}}_{2}B_{12}H_{12}$ dominates the cation distribution, with little evidence of chemical frustration. Even at the shortest distances, where the influence of the anion geometry finally becomes detectable, its symmetry is broadly compatible with the arrangement of occupied trigonal interstitial sites, minimizing the frustration and explaining the lack of fast cation mobility in this phase. Collectively, results like those in figure 3 suggest it is possible to quantify the symmetry mismatch between the polyatomic anion and the lattice packing in order to provide a new descriptor for superionic conductivity in hydroborates and other systems with complex anions.

## 3. Structural frustration

### (a) Cation ordering and site preference

The competition between the local and global environment preferences of the mobile species can have profound effects on the interstitial site occupancy of the cation sublattice, as can other factors such as ion size, temperature, stoichiometry and crystal structure. In superionic materials, these factors can result in having multiple types of available sites occupied with similar probability and no clear preference (as in the flattened energy landscape of figure 1*c*) [23,57,58]. This landscape in turn has significant entropic consequences by increasing the number of configurations explored by the mobile sublattice. The increased availability of accessible sites also ensures that open neighbour sites can be found nearby as a prerequisite for cation hopping.

**Figure 4.**
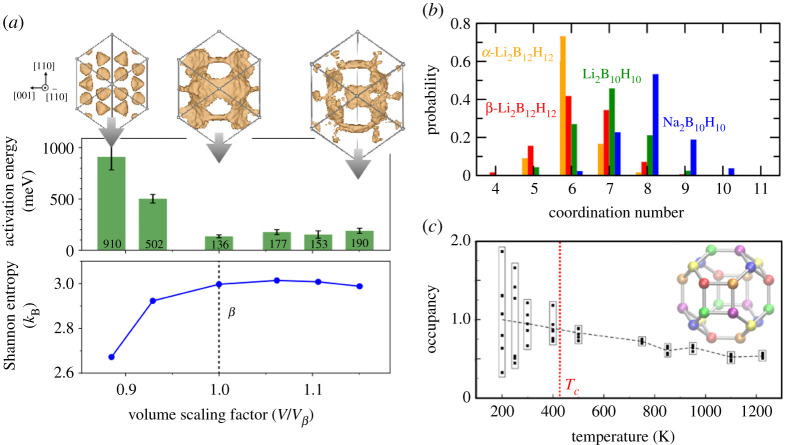
Structural frustration via ordering and site preference. (*a*) Middle: activation energy at 800 K as a function of volume for compressed and expanded superionic $\text{β-}{\mathrm{Li}}_{2}B_{12}H_{12}$. Top: corresponding projected isosurfaces of average ${\mathrm{Li}}^{+}$ density at standard, compressed and expanded volumes. Bottom: the Shannon entropy associated with ${\mathrm{Li}}^{+}$ occupancy probability spread across linear, trigonal and tetrahedral *FCC* sites (see equation (3.1)). Whereas the equilibrium superionic volume shows little site preference (centre), altering the volume enhances the preference for ordering on different sites, slowing diffusion. (*b*) Broad distribution of instantaneous local cation coordination numbers surrounding each anion in select *FCC* *closo*-borate superionic conductors ($\text{β-}{\mathrm{Li}}_{2}B_{12}H_{12}$, ${\mathrm{Li}}_{2}B_{10}H_{10}$, ${\mathrm{Na}}_{2}B_{10}H_{10}$), compared with the narrow distribution for non-superionic $\text{α-}{\mathrm{Li}}_{2}B_{12}H_{12}$, at 800 K. The breadth of the distributions indicates a weak preference for ordering into well-defined coordination environments. (*c*) Temperature-dependent average relative ${\mathrm{Ag}}^{+}$ occupancy of the six unique sets of tetrahedral interstitial sites in the conventional cubic cell of α-AgI, shown as colours in the inset. Unity occupancy represents the limit of even cation distribution across all tetrahedral sites. The dotted line tracks the average value. Near the experimental transition temperature $T_{c}$, the ${\mathrm{Ag}}^{+}$ ions transition from a symmetry-broken ordered configuration to a disordered configuration with no clear occupancy preference. Portions of (*a*) and (*b*) were adapted with permission from Varley *et al*. [20] (Copyright 2016 American Chemical Society) and Kweon *et al*. [21] (Copyright 2017 American Chemical Society). (Online version in colour.)

In figure 4, we illustrate examples of this type of ‘structural frustration’. It is well known that the volume of the crystal lattice and sizes of the anion and cation play a role in determining the preferred interstitial site occupancy of ionic crystals, with foundations in Pauling’s Rules [59]. This effect can be systematically explored in simulations by explicitly changing the volume. As shown in figure 4*a* and discussed at length in previous studies by our group and others [6,11,20,21,60], volume has a drastic influence on both the site occupation and resulting diffusivity of the mobile cation species.

Isosurfaces of cation probability density in the *closo*-borate system ${\mathrm{Li}}_{2}B_{12}H_{12}$ are shown in figure 4*a* for three different unit cell volumes: the equilibrium volume of the superionic β phase (centre), a compressed volume and an expanded volume [21]. It is immediately clear from visual inspection of the isosurfaces that for the equilibrium superionic volume, the cation occupancy spans multiple interstitial sites in a broad interconnected pathway. Indeed, this manifests as the most facile case for ${\mathrm{Li}}^{+}$ hopping from the low activation energy and high diffusivity. However, as volume is decreased or increased, this broad distribution collapses into more discrete site preferences. Accordingly, the configurational entropy is reduced, and cation motion either slows or saturates. Effects of intermediate expansions or contractions are also shown in figure 4*a* and follow the same trend. It is particularly notable that volume expansion does not lead to increased ionic conductivity as would be naively assumed. Instead, any benefit is negated by the reduction in configurational entropy across the available interstitial sites.

A suitable descriptor for the configurational disorder across the interstitial lattice sites can be evaluated by first assigning each cation to its nearest tetrahedral, trigonal or linear interstitial site, then considering the overall occupancies of each interstitial site sublattice (a more detailed analysis can be found in Kweon *et al*. [21]). At the lowest simulated volume of ${\mathrm{Li}}_{2}B_{12}H_{12}$ (which also corresponds to the volume of the non-superionic α-phase), the trigonal site sublattice is 22% occupied, compared with a low 3% occupancy for the other two interstitial site sublattices. At the highest volume, the linear site sublattice is weakly dominant with 15% occupancy, compared to 9% and 11% for the tetrahedral and trigonal sites, respectively. The sublattice occupancies are closest at the equilibrium volume of the superionic β-phase of ${\mathrm{Li}}_{2}B_{12}H_{12}$, standing at 14%, 14% and 10% for the tetrahedral, trigonal and linear sites, respectively—a state of near-maximal entropy representing the structural frustration between these equally likely configurations.

The time-averaged fractional sublattice occupancies $P_{i}$ can be used to further quantify the Shannon configurational entropy S as follows: [21]

3.1 $$S=-k_{B}\sum_{i}f_{i}[P_{i}\ln P_{i}+(1-P_{i})\ln(1-P_{i})].$$

Here, the index i runs over each type of occupied interstitial site (in this case, tetrahedral, trigonal and linear), and $f_{i}$ represents the fraction of total occupied interstitial sites that are of type i. The evolution of the Shannon configurational entropy with volume for ${\mathrm{Li}}_{2}B_{12}H_{12}$ is shown in the bottom panel of figure 4*a*. The trend passes through an entropy maximum and mirrors the activation energy behaviour, with higher entropy (and hence higher structural frustration) corresponding to higher ion mobility.

A corollary of site occupancy can be found in the statistics of local cation coordination around each anion, which acts as a secondary indicator of how ordered the cations are in the interstitial sites [3,21]. In figure 4*b*, we show the distribution of cation coordination numbers around each anion across four *closo*-borates that exhibit *FCC* anion packing, including the Li- and Na-containing ${\left[B_{10}H_{10}\right]}^{2-}$ salts. In the superionic $\text{β-}{\mathrm{Li}}_{2}B_{12}H_{12}$ and ${\left(\mathrm{Li}/\mathrm{Na}\right)}_{2}B_{10}H_{10}$ materials, the distribution of coordination environments is extremely diffuse, representative of cations occupying different interstitial site environments and disrupting the local ordering onto a single sublattice. On the other hand, the non-superionic $\text{α-}{\mathrm{Li}}_{2}B_{12}H_{12}$ phase shows a narrow distribution, with little deviation from expected anion coordination of six. We conclude that the breadth of the distribution of coordination environments is an indicator of superionic behaviour.

Temperature also plays a key role in structural frustration, determining the degree to which the variability in interstitial site occupancies manifests as a key contributor to the configurational entropy. An example of this is shown in figure 4*c* for the prototypical α-AgI superionic conductor, based on results originally reported in Wood & Marzari [13]. The predominant occupied interstitial lattice sites in α-AgI are tetrahedral sites, which form a network through the *BCC* iodide sublattice (see inset of figure 4*c*) [2,13–15]. In the conventional cubic cell, these tetrahedral sites can be further divided into six unique sublattices onto which the cations can be patterned with minimal Coulomb repulsion, the individual occupancy of which can be tracked over the course of the simulation dynamics for superionic, supercooled and superheated α-AgI variants. Below the experimental structural transition temperature to the superionic phase ($T_{c}=420\;K$), the different tetrahedral sublattices exhibit a spread in their occupancies, indicating a global ordering tendency into high- and low-occupancy sublattices. However, as the temperature exceeds $T_{c}$, the available interstitial sites approach uniform occupation with no clear preference, and the ordering tendency is disrupted. This occupation is a clear signature of a thermally driven order–disorder transition that notably appears upon supercooling even in the absence of the low-temperature structural phase transition, which is explicitly suppressed in the simulations. Note also that the average tetrahedral occupancy itself decreases with temperature, indicating a reduced clear preference for these sites over competing octahedral and trigonal sites.

Overall, the temperature-dependent results for α-AgI in figure 4*c* echo the volume-dependent results for ${\mathrm{Li}}_{2}B_{12}H_{12}$ in figure 4*a*, highlighting both the importance of having multiple interstitial sites available—a common feature across several classes of superionic conductors[24]—and the ability to occupy multiple types of sites without a clear energetic preference. Nevertheless, we caution that the availability and occupation of multiple site types does not universally imply easy exchange of ions between those sites. A prime counterexample can be found in the recent work by Morgan on the argyrodites, in which it was shown that the topological connectivity between sites is an equally important factor in determining whether structural frustration from site multiplicity can be leveraged for ion conduction [61].

In addition to volume and temperature [62], other factors can also have a profound influence on structural frustration by preventing cation ordering. One of these is cation off-stoichiometry, which can be induced by doping or alloying. An excellent example of this can be found in the study of Kozinsky *et al.*, who explored the role of ${\mathrm{Li}}^{+}$ stoichiometry in ${\mathrm{Li}}_{7-x}{\mathrm{La}}_{3}{\mathrm{Zr}}_{2}O_{12}$ (LLZO) [63]. The authors found that those intermediate compositions that were fundamentally incompatible with ${\mathrm{Li}}^{+}$ ordering on the interstitial site sublattice were also found to exhibit the fastest diffusion.

### (b) Site distortion and anion packing

Another form of structural frustration can arise from the geometric distortion of the lattice sites themselves. This can arise intrinsically due to anion packing arrangements or from chemical modifications that introduce localized strain fields within the bulk material or at interfaces. Distorted local environments have been cited as motivators for frustration in anti-perovskite ion conductors [1]. Kim *et al.* surveyed a number of different anti-perovskite compositions to show that the energy barrier correlates with site distortion [64]. Other studies investigated alloying as a way to induce site distortion in anti-perovskites. For instance, Effat *et al.* attributed the faster conduction of fluorinated ${\mathrm{Li}}_{2}\mathrm{OHCl}$ in part to distortion of sites upon substitution of $F^{-}$ for ${\mathrm{Cl}}^{-}$, which flattens out the energy minima in the landscape [65]. Chen *et al.* found the same distortion-induced frustration effect could be prompted by incorporating ${\mathrm{Br}}^{-}$ on the ${\mathrm{Cl}}^{-}$ site in ${\mathrm{Li}}_{3}{\mathrm{OCl}}_{1-x}{\mathrm{Br}}_{x}$ [66]. Our own work on these systems has further confirmed the importance of distortion, demonstrating that vacancy-induced distortions in ${\mathrm{Li}}_{3}\mathrm{OCl}$ lead to more frequent jump attempts [67]. Similarly, we found that the alloyed halide conductor ${\mathrm{Li}}_{3}{\mathrm{InBr}}_{6-x}{\mathrm{Cl}}_{x}$ exhibits microstrain distortion effects in the vicinity of the anions and at nanophase boundaries that lead to increased structural frustration [36].

Site distortion can also derive directly from the anion lattice packing geometry [1]. A prime example can be found in the recent study by Di Stefano *et al.*, which introduces the thiophosphate ${\mathrm{LiTi}}_{2}{\left({\mathrm{PS}}_{4}\right)}_{3}$ as an ionic conductor with extraordinarily high ${\mathrm{Li}}^{+}$ mobility [58]. The authors attributed the frustrated energy landscape to a distortion of regular tetrahedral and octahedral sites in the intrinsic lattice. This results in off-centre ${\mathrm{Li}}^{+}$ occupancy, replacing the more symmetric coordination environment in some of the less diffusive thiophosphate variants. In addition, Wang *et al.* performed a broader study on thiophosphates to show that in these systems, lattice packing arrangements with *BCC* symmetry are more effective for facilitating ion mobility [68]. The authors attributed the behaviour to the availability of tetrahedral sites and their topological interconnectedness via face sharing. A secondary factor may be that *BCC* lattices feature intrinsically distorted tetrahedral sites in contrast with the ideal tetrahedral site symmetries in *FCC* structures.

Lattice packing geometries with a higher number of available coordination sites around each anion, including *BCC* lattices, can also have a lower relative electrostatic penalty for temporary fluctuations in local coordination. In particular, the energetic penalty for formation of locally undercoordinated or overcoordinated cation environments around anions in these systems should be reduced because the anion charge is effectively shared with more cation neighbours. We previously quantified the local coordination fluctuations in *FCC* $\text{α-}{\mathrm{Li}}_{2}B_{12}H_{12}$ and *BCC* ${\mathrm{Na}}_{2}B_{12}H_{12}$ in the *closo*-borate systems [21]. These two superionic materials, which have similar diffusion barriers, exhibit similar standard deviations in the local cation coordination around an anion (0.88 for ${\mathrm{Na}}_{2}B_{12}H_{12}$ versus 1.14 for ${\mathrm{Li}}_{2}B_{12}H_{12}$). However, the average total cation coordination around an anion in *BCC* ${\mathrm{Na}}_{2}B_{12}H_{12}$ is much higher (8.4 versus 5.9), meaning the coordination fluctuations have comparatively less electrostatic impact from a bond-valence perspective.

Despite potential intrinsic advantages to packing arrangements such as *BCC*, it is notable that our own survey of multiple *closo*-borate systems did not find any clear correlation between anion lattice packing geometry and barrier [20]. This implies that any such correlation is composition-specific and limited to certain families of superionic conductors. Nevertheless, some recent studies on sodium *closo*-borates in particular have successfully relied on anion mixing as a strategy to improve room-temperature ionic conductivity by preserving *BCC* and other high-symmetry structures at lower temperatures [69,70]. Further research is recommended to clarify the circumstances under which anion lattice packing is likely to impact frustration and to determine the limits of its use as a universal descriptor. One example of a competing factor—already discussed in the context of chemical frustration—occurs when the lattice packing symmetry contrasts with the geometry of a polyatomic anion.

Finally, we point out that the disordered mixing of anions in solid solutions and alloys on an otherwise static lattice can also create an energy landscape that is intrinsically structurally frustrated and unable to accommodate cation coordination preferences. This phenomenon has been extensively cited in the context of the argyrodites [37,61,71–82]. In these systems, structural disorder between the sulfur and halide anions can lead to disruption of the local cation coordination environment and hence increased diffusivity. It is worth noting that in this case, the structural effect supersedes expectations based on the direct cation–anion interactions: while ${\mathrm{Li}}_{6}{\mathrm{PS}}_{5}I$ features higher polarizability and larger lattice volume, ${\mathrm{Li}}_{6}{\mathrm{PS}}_{5}\mathrm{Cl}$ demonstrates higher conductivity because ${\mathrm{Cl}}^{-}$ and $S^{2-}$ sites can disorder. The different valences and ion sizes of the mixed anions, combined with the lack of any regular patterning, leads to an inability of cations to order on the lattice. A very similar phenomenon has also been observed in anode materials, including work by Griffith *et al.* on the niobium tungsten oxides ${\mathrm{Nb}}_{16}W_{5}O_{55}$ and ${\mathrm{Nb}}_{18}W_{16}O_{93}$ [83]. In these materials, Nb and W polyhedral arrangements are intrinsically topologically frustrated, thereby flattening the energy landscape and facilitating ${\mathrm{Li}}^{+}$ diffusion while simultaneously preventing otherwise detrimental structural rearrangements. This avenue is ripe for further study to determine the best compositions for maximizing structural frustration.

## 4. Dynamical frustration

Our analysis so far has focused on sources of frustration that manifest spatially in a static representation of the material, whether through local anion–cation interactions or intrinsic symmetry incompatibilities. However, similar frustration factors can also manifest temporally, through thermal motion that momentarily creates different local physical driving forces. If these forces fluctuate at timescales relevant to cation diffusion, then the competition can prevent ordering and even impart a degree of momentum transfer that can aid mobility. In this section, we consider two categories of ‘dynamical frustration’: anion rotational disorder and activated correlated motion. Each can be signalled by the formation of spatiotemporal ‘hot spots’, for which diffusive events cascade rapidly over relatively short timescales.

### (a) Soft modes and rotational disorder

In typical solids, diffusion occurs far more slowly than the typical optical phonon frequencies. However, if cation diffusion is unusually fast (as in superionic solids), then the soft modes associated with the anion lattice can sometimes perturb the potential energy surface on timescales relevant for diffusion. This effect, discussed widely in the literature, can create momentarily favourable conditions for mobility and induce another source of dynamical frustration [84]. It can be further assumed that the frequency of the perturbations defines a Goldilocks effect to maximize the frustration. If the energy landscape changes too quickly, then the diffusing cations will see only the average effect of the anion dynamics. On the other hand, if the energy landscape changes too slowly, then the diffusing cations will traverse a landscape that is essentially fixed within their dynamical reference frame, receiving no benefit from the fluctuations [21]. At intermediate frequencies, it is further possible to couple the host lattice dynamics directly to the cation motion. A good example can be found in a recent study on α-AgI by Brenner *et al.*, in which the authors were able to conclusively verify a strong connection between ${\mathrm{Ag}}^{+}$ diffusivity and the relaxational motion of the iodide lattice, with anharmonic vibrational effects acting as a key driver of superionic conductivity [85].

The proven connection between lattice softness and cation diffusion has prompted a growing body of literature on the topic. For instance, the lowest optical phonon frequency and similar metrics of lattice softness have found success as possible descriptors for superionic conductivity [1,3,6,9,17,23,27,86,87]. Of particular note are the studies of Muy *et al.*, who showed that superionic conductors have unusually low frequencies of the ${\mathrm{Li}}^{+}$ phonon band centre compared with the host lattice [27]. This property was later used as a metric to successfully screen ion conductors from thousands of known compounds [9].

Soft phonons also form the basis for a class of superionic conductors having internal rotational degrees of freedom that cause orientational disordering of anions even though the overall crystallographic lattice arrangement is retained. In certain contexts, these materials have been referred to as ‘plastic crystals’ [26,88]. The thiophosphate conductors represent one example of a materials class for which correlation between rotational dynamics and cation diffusion has been intensely investigated. Recently, Zhang *et al.* used a joint experiment–theory approach to demonstrate direct evidence of dynamic coupling of polyanion reorientation to Na mobility in ${\mathrm{Na}}_{11}{\mathrm{Sn}}_{2}{\mathrm{PnX}}_{12}$ (Pn=P,Sb; X=S,Se) [89]. They found that ${\left[{\mathrm{PX}}_{4}\right]}^{3-}$ rotation transiently widens the bottlenecks in the migration pathways, introducing dynamical frustration that facilitates ${\mathrm{Na}}^{+}$ diffusion. A similar mechanism has also been suggested in crystalline thiophosphates by several other authors, generally based on correlation between enhanced ${\left[{\mathrm{PX}}_{4}\right]}^{3-}$ rotational mobility and cation diffusion upon chemical or structural modification [84,89–94]. In addition to the crystalline phases, Smith *et al.* demonstrated that significant coupling between cation diffusion and the anion motion can be present in the ${\mathrm{Li}}_{3}{\mathrm{PS}}_{4}$ glass at low temperature [95]. The authors reported a strong overlap between Li vibrational modes and ${\left[{\mathrm{PS}}_{4}\right]}^{3-}$ librational modes in the power spectrum, as well as similar activation energies for anion rotation and Li translation, suggesting that the cation mobility can be enhanced by fostering anion librations in the glass.

Another excellent example of rotationally driven dynamical frustration can be found in the aforementioned *closo*-borate materials. Whereas earlier sections discussed these systems in terms of their static anion geometry and lattice packing, it is also well known that the large anions rotate and reorient. Experimentally, a rapid increase in the reorientation frequency has been linked to the onset of diffusion and the superionic transition, suggesting that diffusion is motivated in part by the order–disorder transition in the anion orientations [48,49,96–100]. A similar correlation has been recorded in multiple simulation studies of these materials systems [20,21,101–104]. Our own investigations showed that the cation vibrational and anion librational frequency ranges overlap, further suggesting the possibility of direct momentum transfer in these systems [21].

**Figure 5.**
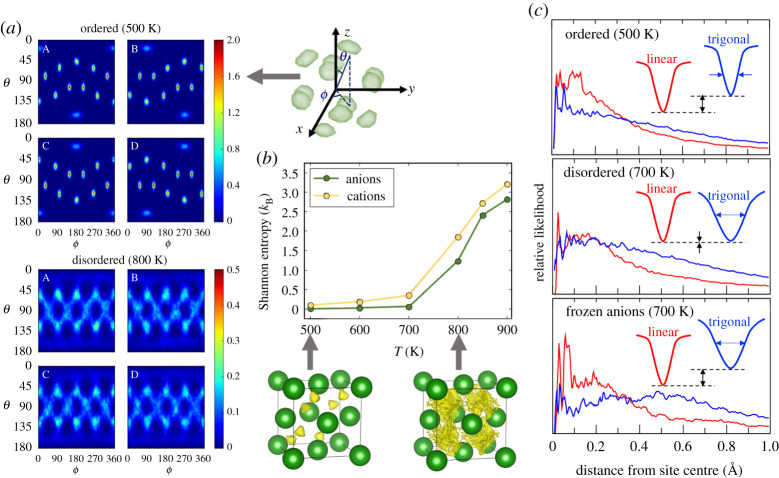
Dynamical frustration via anion rotation. (*a*) Time-averaged orientations of B–H bonds within ${\left[B_{12}H_{12}\right]}^{2-}$ anions of ${\mathrm{Li}}_{2}B_{12}H_{12}$ in lattice polar coordinates. The upper and lower sets of panels are for temperatures below (500 K) and above (800 K) the order–disorder transition temperature for rapid anion reorientation, respectively. The spatial arrangement of the highest-probability ordered orientations and coordinate scheme are shown at right. The four panels in each set represent the four possible sublattices of ordered anion orientations, which are symmetry-broken and ordered below the transition temperature but symmetry-equivalent and disordered above it. (*b*) Temperature-dependent Shannon entropy values (see equation (3.1)) associated with the diversity of cation interstitial site occupations (pale yellow) and of anion orientations (dark green) in Li_2_B_12_H_12_, evidencing the coupled order–disorder transition. Corresponding isosurfaces of cation density (transparent yellow) are shown in the schematics below, with the complex anions rendered as spheres for simplicity. (*c*) Distributions of cation distances from ideal linear and trigonal interstitial site centres below (500 K, top) and above (700 K, middle) the order–disorder transition. The bottom panel is for a 700 K simulation with the anion rotations inhibited, which resembles the low-temperature distribution and features little cation mobility. The insets show schematics of the potential energy wells associated with the two sets of interstitial sites. (Online version in colour.)

Figure 5 presents new analyses that more directly correlate the dynamical fluctuations in the anion orientations with cation motion, as demonstrated for the ${\mathrm{Li}}_{2}B_{12}H_{12}$ archetype. To understand the effect of the order–disorder transition, we consider both temperatures below (i.e. supercooled) and above the onset of superionic behaviour. Figure 5*a* shows a heat map of the anion orientation probabilities within these two temperature regimes. Below the superionic transition, anion reorientations are effectively turned off, and anions naturally order into four sublattices with differently patterned B–H orientations (represented by the four upper panels in figure 5*a*). Above the superionic transition, anion reorientations are activated, and each anion explores all four discrete patterns with equal probability. In this way, the symmetry-breaking ordered behaviour at lower temperatures is superseded by the more entropically favourable disordered configuration. Note this behaviour represents the rotational anion analogue to the cation order-disorder transition discussed for α-AgI in figure 4*c*.

Although the thermal activation of entropically favourable anion orientational disordering in ${\mathrm{Li}}_{2}B_{12}H_{12}$ is clear, the connection of this anion order-disorder transition to the entropically favourable cation diffusive disordering cannot be discerned from the analysis in figure 5*a*. However, tracking the cations shows that they similarly order at low temperature into discrete sites, which become smeared out above the superionic transition (see bottom centre figures of figure 5*b*). The probability densities for anion orientation and cation interstitial site occupation can be discretized (see Methods for details), which allows us to employ equation (3.1) to evaluate the Shannon entropy associated with the exploration of configurations. The results, shown in figure 5*b*, evidence the close correlation between the cation and anion disordering and show a clear transition to superionic behaviour above 700 K. We emphasize that this signature is seen even in the absence of any first-order structural phase transition, which is explicitly suppressed in this set of simulations.

A more direct measure of the effects of anion dynamics on cation mobility and the potential energy landscape can be found by explicitly freezing the degrees of freedom of the anion. Figure 5*c* explores this concept by examining the distributions of ${\mathrm{Li}}^{+}$ distances from linear and trigonal site centres in fully mobile ${\mathrm{Li}}_{2}B_{12}H_{12}$ at temperatures above and below the order–disorder transition, as compared to the frozen-anion case at the hotter temperature. Because the shapes of these distributions are qualitatively representative of the potential energy wells associated with each interstitial site, they can provide a guide for understanding the effect of anion reorientation and temperature on the energy landscape (see schematic insets in each panel of figure 5*c*). Below the transition temperature, the ordered phase shows a narrower and deeper well for the linear site compared to the trigonal site, in agreement with the observed cation distribution. At higher temperature, the depths of these site wells become similar, indicating a loss of clear site preference as discussed in the section on Structural Frustration. However, if anion rotations are disabled, the well associated with the linear site deepens and narrows, in much closer agreement with the low-temperature ordered behaviour. This translates to cation ordering on the linear sites because no anion rotations are available to dynamically alter the energy landscape and fluctuate between trigonal and linear site occupation preferences.

Finally, we point out that anion rotational disorder and highly concerted local cation mobility operate in tandem within the so-called ‘paddlewheel mechanism’, which has been widely discussed in the literature [72,88,93,95,105–112]. Although this term is sometimes invoked to describe any type of anion rotation dynamical frustration, in our view this definition is too liberal. Instead, we propose that the paddlewheel should represent a specific subset of dynamical correlation in which cations are passed in a concerted manner among rotating anions through direct momentum transfer. In this context, anion rotation is a necessary but not sufficient condition: its role is not merely to corrugate the energy landscape, but also to drive correlated cation dynamics. In practice, direct momentum transfer is much more difficult to detect than simple correlation. However, the excellent study by Zhang *et al.*, on ${\mathrm{Na}}_{11}{\mathrm{Sn}}_{2}{\mathrm{PX}}_{12}$ represents an important step towards affirming the existence of the paddlewheel effect in that system [89], as do the studies of Smith *et al.*, on glassy ${\mathrm{Li}}_{3}{\mathrm{PS}}_{4}$ [95] and our own work coupling cation vibrational and anion librational frequency ranges for *closo*-borates [21].

### (b) Correlation and local fluctuations in cation mobility

Despite conventional wisdom that superionic solids feature liquid mobile sublattices, a more accurate description is that the mobile sublattice exhibits behaviour that is hybrid between a liquid and a solid [2,113]. Specifically, instead of all ions being uniformly fluid, some superionic materials can exhibit spatiotemporal ‘hot spots’ with cascading dynamical events occurring together over short timescales. Averaged over long times, this behaviour approaches the ergodic limit. However, at shorter times, there exists a broad range of spatiotemporally local mobilities, which leads to instantaneous coexistence of both fast and slowly diffusing ions—effectively, liquid and frozen cation sublattices, respectively. Regions of the material may therefore continually fluctuate between these two limits, activating and deactivating diffusion dynamically (analogous to local density fluctuations near a critical point).

**Figure 6.**
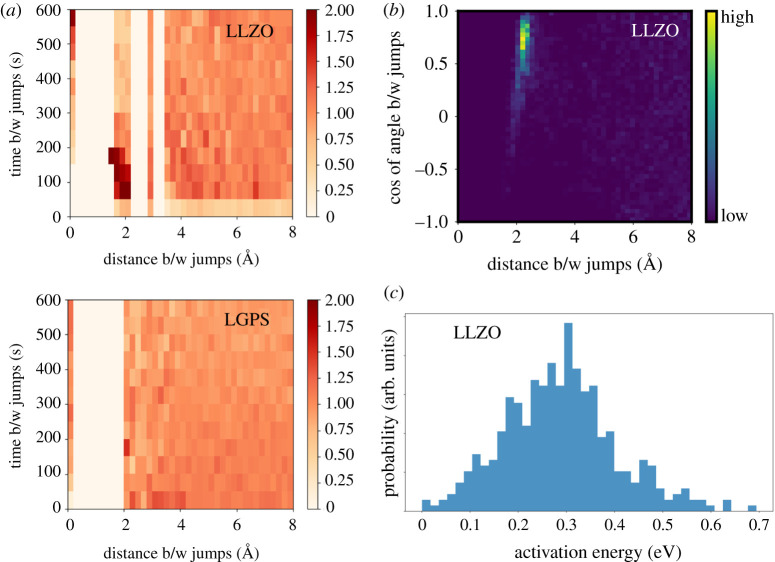
Dynamical frustration from local fluctuations in cation sublattice mobility. (*a*) Spatiotemporal distribution of cation jump events in LLZO and LGPS. Jump intervals and distances with high probability (darker colour) represent diffusive hot spots. In each case, the data are normalized against the uncorrelated limit at long time intervals. Jumps in LLZO show strong correlations and hot spots lasting 50–200 fs in duration, whereas LGPS shows highly random jumps with only weak correlations in space and time. (*c*) Distribution of angles between vectors of discrete ${\mathrm{Li}}^{+}$ jumps in LLZO occurring within 140 fs as a function of distance between the jump sites, showing the high degree of directional correlation. (*d*) Distribution of diffusion constants for individual diffusing ions in uncorrelated segments of LLZO dynamics, evidencing the breadth of local diffusive timescales. (Online version in colour.)

From a practical standpoint, the existence of dynamical hot spots is connected to high degrees of correlation in the jump behaviour [3,10,12,19,114,115]. Figure 6 shows the importance of correlated dynamics for the garnet ion conductor LLZO, which has been discussed in several previous studies [115–121]. We first discretize the jump events (see Methods), then introduce a spatio-temporal jump analysis that quantifies the correlations between jumps separated in space and time (figure 6*a*). The values are normalized by the average over time at each distance, so values above 1.0 show a higher probability for correlated jumps for a given time and distance. For LLZO, diffusive hot spots are clearly visible, extending out to one or two nearest-neighbour sites, with correlated jumps occurring within 50–300 fs. In LLZO, these jumps are also highly directionally correlated, as shown in the analysis in figure 6*b*.

Such correlated diffusive behaviour—both in space and time—was also explored by Morgan and Madden for AgI [12] and more recently by He *et al.* across a variety of lithium conductors [10]. However, we point out that the existence of highly correlated dynamics and hotspots—while common in many solid electrolytes—is not itself a necessary condition for superionic behaviour. This can be seen by comparing the spatiotemporal analysis of LLZO to that of the thiophosphate ${\mathrm{Li}}_{10}{\mathrm{GeP}}_{2}S_{12}$ (LGPS) [60,93,122–125] in figure 4*a*. In contrast to LLZO, LGPS has almost liquid-like anisotropic diffusion along one-dimensional channels, with little clear evidence of hotspots. Although the cation motion remains highly correlated [10], there is no particular timescale associated with hotspots; instead, these correlations appear mostly randomly in the time domain.

The ability to track ions independently in the simulations also permits direct statistical analysis of the variability in cation diffusivities. Whereas the ensemble-averaged mean-squared displacement gives rise to a single mean diffusion coefficient, we can instead track each individual ion over a range of uncorrelated simulation frames and compile the results into a single distribution. This is demonstrated for LLZO in figure 6*c*, where we have used linear fits to Arrhenius data for each ion to extract the distribution of individual activation energy barriers over the course of the full simulation. The barriers show a surprisingly broad range from ultrafast, liquid-like flow ($E_{a}<0.1\;\mathrm{eV}$) to very slow, solid-like hops ($E_{a}$ approaching 0.7 eV). The long tail at higher activation barriers is particularly notable, since it represents local spatiotemporal zones that are effectively frozen [126]. Sites in this region are symmetrically identical to the fast-diffusing sites, yet dynamical fluctuations in the cation or anion sublattice cause some regions to be temporarily far more conducive to ion hopping than others. We have explored this coexistence of fast and slow conduction modes and its implications for dynamical frustration in some of our previous work [20,127].

## 5. Conclusion

We have drawn upon examples from molecular dynamics simulations of a wide variety of superionic conductors to illustrate chemical, structural and dynamical sources of frustration and their connection to rapid ion mobility. We have combined discussions of literature references with new analyses of our own studies of halide, oxide, sulfide and *closo*-borate solid electrolytes to further illustrate the principles that underlie our definitions of frustration. Collectively, these factors act to create competition at the atomic scale, the result of which is to flatten the energy landscape and maximize configurational entropy while retaining the solid structure. In this way, the system is able to balance entropic and enthalpic contributions in the superionic phase.

For chemical frustration, we discussed the role of anion polarization and directional covalent character in the anion–cation bond, which can compete with conventional electrostatic preferences to prevent cation ordering in halides and sulfides. We also provided examples of frustrated competition in *closo*-borates between the lattice symmetry and the geometry of the polyatomic anion, which acts as a template for cation coordination sites.

For structural frustration, we drew upon *closo*-borate conductors to discuss the competition between different interstitial site preferences. We showed that for these materials, no clear site preference exists, and the structure and volume of the solid phase acts to maximize the configurational entropy. We also showed that for α-AgI, thermal effects can activate an order–disorder transition in the cation interstitial site occupancy, removing the symmetry-breaking site ordering preference that dominates at lower temperatures. Additional discussions of structural frustration due to site distortion and anion lattice packing were also invoked, drawing primarily upon previously published examples.

Finally, for dynamical frustration, we discussed the role of anion rotation in *closo*-borates, which creates fluctuations in the potential energy landscape that momentarily favour cation rearrangement. Correlated jump events in oxides and halides were also discussed in the context of diffusive hot spots, which activate and deactivate in the course of the dynamics. Hot spots paint a picture in which cations do not traverse a fixed energy landscape but instead respond to a dynamically changing landscape.

As indicated throughout this paper, similar ideas of frustration have been widely discussed in the literature, often using different nomenclature. We propose that our categorizations, originally introduced in the context of *closo*-borates [21], can be adopted by the community as a universal language to describe how frustration leads to low activation barriers in superionic materials. As a first step towards this goal, table 1 provides a summary of key families of cation-conducting solid-state electrolytes for which concepts resembling our descriptions of frustration have been specifically identified in previous work. In each case, we attempt to classify these reports within the terminology defined here, with accompanying citations to the original articles.

**Table 1.** Examples of frustration in some known solid electrolytes.

material system	examples	probable drivers
LPS-type thiophosphates	${\mathrm{Li}}_{3}{\mathrm{PS}}_{4}$, ${\mathrm{Li}}_{2}S\text{–}P_{2}S_{5}$, ${\mathrm{Li}}_{7}P_{3}S_{11}$, ${\mathrm{Li}}_{4}P_{2}S_{6}$, ${\mathrm{Li}}_{3.25}{\mathrm{Si}}_{0.25}P_{0.75}S_{4}$, ${\mathrm{Na}}_{3}{\mathrm{PS}}_{4-x}{\mathrm{Se}}_{x}$	*chemical:* substitution for P or S changes bonding, polarizability and local occupancy [84,128,129]
		*structural:* destabilized energy minima in amorphous phase due to lack of crystalline order [95]; structural arrangement and local coordination symmetry of anions impacts diffusivity [130,131]; vacancy-induced site disorder and partial site occupancy induce mobility [91]; atomic substitution can tune lattice volume and relative cation site preference to maximize frustration [132,133]; ion conduction correlates with lack of cation site preference [134]
		*dynamical:* enhanced ${\left[{\mathrm{PS}}_{4}\right]}^{3-}$ reorientations from vacancies, substitutions, or volume changes lead to faster conductivity [84,90–92,95]
LGPS-type thiophosphates	${\mathrm{Li}}_{10}{\mathrm{GeP}}_{2}S_{12}$, ${\mathrm{Li}}_{10}{\mathrm{SiP}}_{2}S_{12}$, ${\mathrm{Na}}_{10}{\mathrm{GeP}}_{2}S_{12}$, ${\mathrm{Na}}_{10}{\mathrm{SnP}}_{2}S_{12}$, ${\mathrm{Na}}_{11}{\mathrm{Sn}}_{2}P_{2}S_{12}$, ${\mathrm{LiTi}}_{2}{\left({\mathrm{PS}}_{4}\right)}_{3}$	*chemical:* inductive effect through S interaction affects barriers [38–40]; substitution with O changes bonding [41]
		*structural:* site occupancies disorder above transition temperature [93]; Ge/P site disorder can increase conductivity [135]; O substitution for S changes site occupancy[41]; distorted intrinsic site symmetry, fluctuations in the coordination environment, and lack of clear site preference enhance frustration in Ti-based variant [58]; conduction pathway changes with local site volume [40]
		*dynamical:* enhanced ${\left[{\mathrm{PS}}_{4}\right]}^{3-}$ reorientations from structural or chemical modification lead to faster conductivity [89,93,94]; highly correlated migration in sulfide and oxygen-substituted variants [10,41]
Argyrodite thiophosphates	${\mathrm{Li}}_{6}{\mathrm{PS}}_{5}X\;(X=\mathrm{Cl},\mathrm{Br},I)$	*chemical:* anion substitution can tune polarizability, cation–anion bond covalency and cation–cation interactions to optimize diffusion [37,71,75,80,136,137]
		*structural:* anion site disordering from anion substitution or vacancy introduction prevents preferred cation ordering and facilitates inter-cage jumping [37,71–82]; cation diffusion occurs via percolating pathways of local disorder characterized by ranges of anion–cation coordinations [61]
		*dynamical:* ${\left[{\mathrm{PS}}_{4}\right]}^{3-}$ reorientations couple to ion conduction [72]
LATP-type phosphates	${\mathrm{LiTi}}_{2}{\left({\mathrm{PO}}_{4}\right)}_{3}$, ${\mathrm{Li}}_{1+x}{\mathrm{Al}}_{x}{\mathrm{Ti}}_{2-x}{\left({\mathrm{PO}}_{4}\right)}_{3}$	*chemical:* delocalization of polarization interactions and competition in cation–anion bonding aids diffusion [138]
		*structural:* arrangement of locally distorted sites is important [138]
		*dynamical:* spatio-temporally correlated ion dynamics [10]
Anti-perovskite family	${\mathrm{Li}}_{3}\mathrm{OX}\;(X=\mathrm{Cl},\mathrm{Br},I)$, ${\mathrm{Na}}_{3}\mathrm{OX}$, ${\mathrm{Li}}_{3}O_{0.5}S_{0.5}I$, ${\mathrm{Li}}_{3}{\left(\mathrm{OH}\right)}_{2}X$, ${\mathrm{Li}}_{2}\mathrm{OHCl}$	*chemical:* Vacancy–vacancy interactions through polarizable anions cause local structural disorder [67]; vacancy-metal dopant interactions affect diffusion barrier [139]
		*structural:* distorted local environments from defects or alloying lower diffusion barrier [64–66,140]
		*dynamical:* rotations of cation coordination complexes around oxide and hydride anions correlates with fast diffusion [65,105,141,142]
Hydroborates and *closo*-borates	${\left(\mathrm{Li}/\mathrm{Na}\right)}_{2}B_{12}H_{12}$, ${\left(\mathrm{Li}/\mathrm{Na}\right)}_{2}B_{10}H_{10}$, $(\mathrm{Li}/\mathrm{Na}){\mathrm{CB}}_{11}H_{12}$, ${\mathrm{LiHCB}}_{11}H_{5}{\mathrm{Cl}}_{6}$, $\mathrm{Na}({\mathrm{CB}}_{11}H_{12})(B_{12}H_{12})$, ${\mathrm{Na}}_{2}(B_{12}H_{12})(B_{10}H_{10})$, ${\mathrm{LiBH}}_{4}$	*chemical:* symmetry incompatibility between anion geometry and lattice arrangement [21,56,100]; incorporation of polarizable or aliovalent atoms within complex anions alters local bonding [22,48,49,99,104,143]
		*structural:* anion mixing introduces geometric frustration and site disorder that enhance ion conduction [52,69,144,145]; lack of site preference or saturation of low-energy sites creates frustration [21,48,104]
		*dynamical:* reorientations of complex anions couples to cation diffusion [21,48,49,97,98,100–104,146]; spatiotemporal hot spots in correlated diffusion [20]
Ag/Cu halides & chalcogenides	AgI, CuI, ${\mathrm{Ag}}_{0.5}{\mathrm{Cu}}_{0.5}\mathrm{Br}$, ${\mathrm{Ag}}_{2}X$ (X=S,Se), ${\mathrm{Cu}}_{2}X$	*chemical:* frustration between covalent local coordination environment and lattice geometry promotes ion conduction [13,34,114,147–149]
		*structural:* anharmonic relaxation of anion lattice couples with cation diffusion [85]; diffusion linked to range of competing coordination environments [149]; deviations from stoichiometry prevent cation ordering and promote diffusion [150]
		*dynamical:* spatio-temporally correlated motion can lower the activation energy barrier [12,114,149]; direct coupling between lattice dynamics and cation motion [85]
Garnet family	${\mathrm{Li}}_{7-x}{\mathrm{La}}_{3}{\mathrm{Zr}}_{2}O_{12}$, ${\mathrm{Li}}_{5}{\mathrm{La}}_{3}{\mathrm{Ta}}_{2}O_{12}$, ${\mathrm{Li}}_{3+x}{\mathrm{Nd}}_{3}{\mathrm{Te}}_{2-x}{\mathrm{Sb}}_{x}O_{12}$	*chemical:* frustration between local cation coordination environment and lattice symmetry [57]
		*structural:* concentration-dependent ionic conductivity from competing site preference and disruption of global cation ordering [63,118]; local site distortion can create frustration that correlates with cation mobility [57,151]
		*dynamical:* spatio-temporally correlated cation motion via dynamical excitations [10,115,118–121]; enabling rotation of oxygen-containing anions improves conduction [152]
${\mathrm{Li}}_{3}{\mathrm{MX}}_{6}$-type halides	${\mathrm{Li}}_{3}{\mathrm{InX}}_{6}\;(X=\mathrm{Br},\mathrm{Cl})$, ${\mathrm{Li}}_{3}{\mathrm{YX}}_{6}$, ${\mathrm{Li}}_{3}{\mathrm{ErX}}_{6}$, ${\mathrm{Li}}_{3}{\mathrm{LaI}}_{6}$, ${\mathrm{Na}}_{3-x}{\mathrm{Er}}_{1-x}{\mathrm{Zr}}_{x}{\mathrm{Cl}}_{6}$	*chemical:* cation–anion interaction induces bond frustration due to fluctuations in covalent character [35,36]
		*structural:* increased site disorder, altered site connectivity and site symmetry distortion in mixtures and substituted alloys alter transport properties [153–155]; multiplicity of occupation sites enhances mobility [155]
		*dynamical:* softer lattice dynamics with more polarizable halides lowers migration barrier [156]

From table 1, it is clear that no single source of frustration dominates across all systems. This agrees with conclusions from recent unsupervised learning studies by Zhang *et al.* [29] and Kahle *et al.* [25], in which no universal descriptor for lithium superionic conductivity could be identified. Nevertheless, across each of the surveyed families of solid-state electrolytes, there is a combination of closely related frustration factors at play that are derived from similar physical origins. In addition, not all of the sources of frustration coined here are expected to be equally prevalent in all superionic materials. For instance, chemical frustration is likely to be more impactful when anions with high polarizability are present. Some structural frustration mechanisms require well-defined crystallographic lattice sites, whereas others, such as an ability to achieve patterned ordering of cations, are enhanced in glassy conductors. Dynamical frustration via anion rotation requires internal degrees of freedom. Correlated motion requires well-defined conduction channels.

In addition to providing a more robust classification scheme for reported motivations for high ion mobility, our definitions can serve as a basis for categorizing future efforts to improve ionic conductivity by promoting frustration. Several such efforts are already tabulated in table 1. We also hope that the new analyses presented here may prompt the development of descriptors for superionic conductivity associated with each category, which can aid in screening for new solid electrolytes or optimization of existing systems.

Nevertheless, we caution that frustration-induced flattening of the energy landscape is a useful but not sufficient condition for an effective solid electrolyte. Other factors are required in order to ensure rapid long-range ionic conductivity, as have been described at length elsewhere. One critical requirement is the existence of a high density of charge carriers [2,3,157,158]. These can be vacancies or interstitials that are intrinsic to the crystal structure or else incorporated via strategies such as aliovalent doping. Moreover, fast ion conduction requires a percolating network of low-barrier pathways to ensure long-range diffusion [3,7,64,68]. Whereas frustration can help to lower diffusion barriers for local mobility, it does not necessarily guarantee that such topological pathways commonly exist. Diffusion may also be limited to one-dimensional channels, which can be easily blocked. The importance of having a percolating network of low-barrier transitions has been emphasized by Wang *et al.* in the context of sulfide-based conductors [68] and by Morgan in the context of argyrodites [61].

Despite the recent interest in frustration-related mechanisms, many details remain less well understood and should be considered high-priority research directions for future study. One of the most impactful of these concerns amorphous solid electrolytes, which can have significant processing advantages over crystalline variants. Amorphous materials exhibit intrinsic structural frustration by preventing cation ordering, introducing native site distortion and promoting a dispersion of local environments [23]. However, some ion conductors show dramatically reduced ion conductivity upon amorphization, whereas others are unaffected or even enhanced [159–161]. The origins of these differences are unclear, in part due to the dearth of simulation data on glassy systems. A few studies have pursued this direction for LPS-type glasses, finding evidence that topological connectivity [162] or reduced hindrance to rotation [95] may explain the elevated conductivity of these systems in the amorphous phase. Nevertheless, further research is recommended to determine the specific circumstances under which glassy conductors can fully leverage frustration. This could accelerate the development of new descriptors for amorphous conductors.

Another promising area for future research is the coupling between frustration and external stimuli. Although the analysis presented here focuses on intrinsic properties of superionic conductors, these external factors could be used to alter properties in a targeted way. For instance, mechanochemical synthesis pathways have been invoked as a way to extrinsically modify site disorder and structural frustration in solid electrolytes [153,163]. In addition, beyond random (Brownian) fluctuations, dynamical frustration could be driven or enhanced by factors such as electrochemical gradients, strain fields and optical excitation. An intriguing example can be found in the recent report by Gordiz *et al.* describing resonance-driven phonon processes as a means to substantially enhance ion conduction in Ge-substituted ${\mathrm{Li}}_{3}{\mathrm{PO}}_{4}$ [164]. Future efforts along these lines could unlock the potential for dramatic increases in ion mobility by targeting the precise chemical, structural and vibrational factors that are the most promising.

## 6. Methods

### (a) Molecular dynamics

Unless otherwise noted, all AIMD simulations were run using the CP (Car-Parrinello) [165] module within Quantum Espresso [166] using ultrasoft Perdew-Burke-Ernzerhof (PBE) pseudopotentials [167,168] provided from the Quantum Espresso standard pseudopotential library. Gamma-only *k*-point sampling was used. Simulations were run in the *NVT* ensemble, and Nosé–Hoover chains[169] were employed to maintain temperature. Simulation details are provided in previous publications or are summarized here.

Binary metal halides: Car-Parrinello AIMD simulations were run on the superionic α phases of AgI, CuI and CuBr, as well as zinc blende CuCl and rocksalt LiCl. Simulations were run for 35–50 ps of production time using supercell sizes of 32 (CuCl), 64 (LiCl) or 108 (CuBr, CuI, AgI) atoms (except CuBr, which was run for 16 ps). For CuX, a CP fictitious mass of 800 a.u. and a timestep of 0.15–0.2 fs were used. For AgX, a CP fictitious mass of 400 a.u. and a timestep of 0.3 fs were used. For rocksalt LiCl, a single vacancy was introduced in the 64-atom supercell to facilitate hopping, and a CP fictitious mass of 400 a.u. and a timestep of 0.2 fs were used. All simulations were run at 700 K except LiCl, which was run at 800 K. The supercell lattice parameters were a=15.5 Å (AgI), 14.3 Å (CuI), 13.5 Å (CuBr), 8.5 Å (CuCl) and 10.3 Å (LiCl).

*Closo*-borates: Car-Parrinello AIMD simulations were run for 50–60 ps of production time using $\sqrt{2}\times \sqrt{2}\times 1$ supercells of *FCC* phases of $\text{α-}{\mathrm{Li}}_{2}B_{12}H_{12}$, $\text{β-}{\mathrm{Li}}_{2}B_{12}H_{12}$, ${\mathrm{Li}}_{2}B_{10}H_{10}$ and ${\mathrm{Na}}_{2}B_{10}H_{10}$. A CP fictitious mass of 400 a.u. and a timestep of 0.15 fs were used. The mass of hydrogen was set to that of deuterium. The supercell lattice parameters were a=14.21, c=10.05 Å ($\text{β-}{\mathrm{Li}}_{2}B_{12}H_{12}$); a=13.62, c=9.63 Å ($\text{α-}{\mathrm{Li}}_{2}B_{12}H_{12}$); a=13.23, c=9.36 Å (${\mathrm{Li}}_{2}B_{10}H_{10}$); a=14.51, c=10.26 Å (${\mathrm{Na}}_{2}B_{12}H_{12}$); and a=13.96, c=9.87 Å (${\mathrm{Na}}_{2}B_{10}H_{10}$). Lattice-contracted and lattice-expanded variants of ${\mathrm{Li}}_{2}B_{12}H_{12}$ were generated by applying equiaxial volumetric strains corresponding to 12% and 14% contraction and expansion, respectively. Further information can be found in [20,21].

${\mathrm{Li}}_{3}{\mathrm{PS}}_{4}$: Born–Oppenheimer AIMD simulations were run with the Vienna Ab-initio Simulation Package (VASP) [170] using projector augmented wave pseudopotentials [171]. A timestep of 1 fs and a 128-atom supercell for 50 ps of production time were used. The supercell lattice parameters were a=12.78 Å, b=15.74 Å and c=12.04 Å.

LGPS: Car-Parrinello AIMD simulations were run for 45 ps of production time using a $\sqrt{2}\times \sqrt{2}\times 1$ supercell at 700 K. A CP fictitious mass of 400 a.u. and a timestep of 0.15 fs were used. The supercell lattice parameters were a=12.69 Å, b=12.45 Å and c=12.45 Å.

LLZO: Classical molecular dynamics simulations were run on the cubic phase of ${\mathrm{Li}}_{6.25}{\mathrm{La}}_{3}{\mathrm{Zr}}_{2}O_{12}$ using the LAMMPS code [172] based on potentials adopted from Ref. [63]. Production runs were for 5 ns with a 1 fs timestep at 1100 K on a 2×2×2 supercell. A Nosé–Hoover thermostat was employed to maintain the temperature.

### (b) Diffusion barriers

The diffusion activation energy barriers in figures 4*a* and 6*c* were determined by performing linear fits to the long-time behaviour of the cation mean squared displacement at each simulated temperature, then using the Nernst–Einstein relation to extract the diffusion coefficient. Temperature-dependent diffusion coefficients were then fitted to an Arrhenius equation to extract the activation energy barriers. In each case, at least five simulated temperatures were used.

### (c) Maximally Localized Wannier Functions and polarization

MLWFs in figure 2 were calculated for 100 frames of each AIMD simulation using the wannier90 package [173]. Frames were sampled from runs at 700 K, except for LiCl at 800 K. Atomic polarization was computed as the vector sum of the MLWFs associated with each anion.

### (d) Site, jump and orientation identification

For the data in figure 6, sites and diffusive jump events were identified using the sitator [174] software. In figure 6*b*, individual ${\mathrm{Li}}^{+}$ velocity vectors were averaged over a window of 140 fs, and a minimum-velocity cutoff of 16 Å/s was used to isolate ions that were mobile within this window. In figure 4, sites in the *closo*-borate systems and in α-AgI were determined according to the closest interstitial site centre to the instantaneous cation position during the dynamics runs. Discrete orientations of ${\mathrm{Li}}_{2}B_{12}H_{12}$ for the Shannon entropy analysis in figure 5 were assigned by selecting a $20^{\circ }$ window centred on the maxima identified in figure 5*a*.

## Data accessibility

The raw molecular dynamics simulation trajectories for the superionic systems are available via Dryad. Wood, Brandon *et al.* [175].
